# Dietary fiber chemical structure determined gut microbiota dynamics

**DOI:** 10.1002/imt2.64

**Published:** 2022-11-28

**Authors:** Xin Meng, Jun Zheng, Fengqiao Wang, Jie Zheng, Dong Yang

**Affiliations:** ^1^ Beijing Key Laboratory of Functional Food from Plant Resources, College of Food Science & Nutritional Engineering China Agricultural University Beijing China; ^2^ Center for Food Safety and Applied Nutrition U.S. Food and Drug Administration College Park Maryland USA

**Keywords:** *C. elegans*, CAZyme, chemical structure, dietary fiber, glycosidic bonds, microbiota

## Abstract

Precision modulation of gut microbiota requires elucidation of the relation between dietary fiber intake and gut microbe dynamics. However, current studies on this aspect are few due to many technical limitations. Here, we used *Caenorhabditis elegans* to minimize the complicated host–microbial factors and to find the relation between dietary fiber chemical structures and gut microbiota dynamics. The *Allium schoenoprasum* polysaccharide (*As*sP) structure was elucidated and used as the complex dietary fiber against the simple fiber inulin. In vitro bacterial growth and genome analysis indicated that *As*sP supports bacterial growth better than inulin, while in vivo gut microbiota analysis of *C. elegans* fed with *As*sP showed that microbiota richness increased significantly compared with those fed with inulin. It is concluded that the more complex the dietary fiber chemical structure, the more gut bacteria growth it supports. Together with the community bacterial interactions that alter their abundances in vivo, these factors regulate gut microbiota synergistically.

## Highlights

- Chemical structure of a complex dietary fiber *Allium schoenoprasum* polysaccharide (*As*sP) was determined.
- In vitro bacterial growth monitored with *As*sP or inulin as the sole carbon source.
- In vivo gut microbiota dynamics analyzed with *As*sP or inulin fed to *Caenorhabditis elegans*.
- Genomic analysis pointing out bacterial utilization mechanism of carbon source.

## Graphical abstract

The monosaccharide composition and glycosidic bond configuration determine the CAZyme that could hydrolyze it. Thus, bacteria encoding specific CAZyme utilize different structured dietary fibers. Compounded with bacterial interaction, gut microbiota dynamics is synergistically modulated.

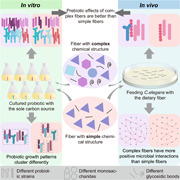

## INTRODUCTION

Dietary carbohydrates are the direct and major calorie source to the host and can be categorized into sugar, starch, and fiber. While dietary carbs in our food are vastly different, our genome only encodes 17 known digestive enzymes for lactose, starch, and sucrose and none for dietary fibers [1]. Dietary fibers are the most structurally diverse polymers, which not only adopt secondary structure but also exhibit crystalline form [2]. Parallel to this, it is suggested that we are carrying a population of microbes harboring more than 100 times greater (than ours) number of genes encoding proteins designed to sense and degrade dietary fibers [3, 4, 5]. Different dietary fiber could induce different gene expression in a bacterium for its utilization [6], and many evidence show that there is a three‐way relationship between gut microbiota, dietary fibers, and the mucus layer [7, 8]. The metabolites of dietary fibers by gut microbiota impact the host's fitness in many aspects, and precise supplementation of dietary fiber is a promising intervention for gut microbiota‐related health issues [9, 10, 11, 12]. Many dietary fibers, natural or artificial, have been studied on their modulation of the gut microbiota including cellulose, pectin, type‐II and type‐IV resistant starches, and complex arabinoxylan [13, 14, 15, 16]. It was initially proposed that the dietary fiber chemical structure was related to the gut microbiota dynamics [17], thus certain chemically structured dietary fibers for specific gut bacteria could reach predictable gut microbiota modulation [18]. Although there are some preliminary reports on the relationship between dietary fiber structure and gut microbiota dynamics, the detailed mechanism behind them remains largely unknown [19].

*Caenorhabditis elegans* has been used as the animal model for many studies and it has been recently suggested as a model for microbiome research, including the host–probiotic relations and the diet micronutrient interactions with commensal microbes [20, 21, 22, 23, 24, 25]. Unlike the complex gut compartmentalization in other animal models (including different parts of the gut, and mucus‐adjacent or luminal communities in the same part of the gut), the simplicity of *C. elegans* gut further helps minimization of host factors affecting the dietary fiber–microbiota relationship determination [26]. Besides, the food simplicity of laboratory cultured worm (typically the *Escherichia coli* OP50 lawn on a nematode growth medium [NGM]) renders supplementation of any dietary fiber an affordable while a significant factor in its diet.

Choice of the dietary fiber (especially water‐soluble ones) has a significant impact on the outcomes and interpretation of dietary effects on host gut microbiota [27]. Inulin has shown significant modulation of the gut microbiota and is used in this study as the simple dietary fiber for its simple structure as β−2,1‐glycosidic bond‐linked fructose [28, 29]. For complex dietary fiber, the major polysaccharide from the stalk of *Allium schoenoprasum* (*As*sP), a commonly seen vegetable and seasoning on the dining table worldwide, the monosaccharide compositions of which we have previously identified, was used [30]. Here, in vitro and in vivo bacteria dynamic studies with inulin or *As*sP as the sole carbon source and bacteria genome analysis were combined to identify the relationship between dietary fiber chemical structures and gut microbiota dynamics.

## RESULTS

### Structural differences between dietary fiber *As*sP and inulin

**Figure 1:**
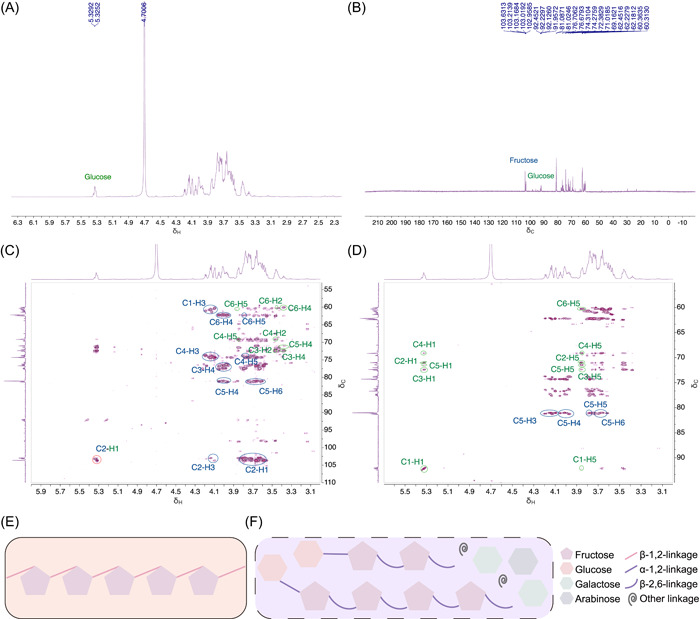
NMR spectrum of deuterated *Allium schoenoprasum* (*As*sP) in D_2_O. (A) One‐dimensional ^1^H spectrum, (B) one‐dimensional ^13^C spectrum, (C) two‐dimensional HMBC spectrum, and (D) two‐dimensional HSQC‐TOCSY spectrum of deuterated *As*sP in D_2_O at 300 K. Green labeling indicates signals of glucose residues and blue labeling indicates signals of fructose residues. (E) Cartoon representation of inulin chemical structure as β−2,1‐glycosidic bond‐linked fructose. (F) Cartoon representation of possible *As*sP chemical structure as α−1,2‐glycosidic bond‐linked glucose and fructose, β−2,6‐glycosidic bond‐linked fructose, and other bond‐linked galactose and arabinoses

We have initially analyzed the monosaccharide composition of *As*sP, and here, we further determined its structure (specifically the glycosidic linkages) by a combination of one‐ and two‐dimensional NMR spectroscopy methods [30]. In the ^13^C NMR spectrum, δ 103.0–103.7 indicates a quaternary carbon and the absence of the terminal hydrogen signal in the ^1^H NMR spectrum indicates a → 6)‐β‐d‐Fruf‐(2 → terminal carbon signal of the fructose residue (Figure 1A,B) [31]. The ^1^H signal at *δ* 3.58–3.84 correlates with the *δ* 103.0–103.7 signal of C‐2 in the HMBC spectrum, indicating the presence of H‐1 (Figure 1C). The signal at *δ* 4.09–4.19 correlates with *δ* 103.0–103.7 of C‐2 and *δ* 61.4 and 60.3 of C‐1 in the HMBC spectrum, indicating the presence of H‐3. The *δ* 4.09–4.19 signal correlates with *δ* 4.05–3.96 in the COSY spectrum, indicating the presence of H‐4 (Supporting Information: Figure S1A). The *δ* 4.05–3.96 signal correlates with *δ* 81.0–81.3 and 62.2 in the HMBC spectrum, indicating the former as C‐5 and the latter as C‐6 (Figure 1C). The ^13^C signal of *δ* 81.0–81.3 correlating with ^1^H at *δ* 4.09–4.19, 3.96‐4.05, 3.78–3.80, and 3.61–3.75 in the HSQC‐TOCSY spectrum further confirmed the β‐d‐Fruf (6 → 2) structure (Figure 1D). Similarly, linkage of α‐d‐Glcp‐(1 → 2)‐β‐d‐Fruf could be identified. The chemical shift identification of each residue was also assisted by the HSQCED, H2BC, and NOESYPHER spectra (Supporting Information: Figure S1B–D) and summarized in Supporting Information: Table S1. It is known that inulin consists of β−2,1‐glycosidic bond‐linked fructose (Figure 1E), and we identified *As*sP as α−1,2‐glycosidic bond‐linked glucose and fructose, β−2,6‐glycosidic bond‐linked fructose, and other bond‐linked galactose and arabinoses (Figure 1F).

### In vitro probiotic growth with *As*sP or inulin as the sole carbon source

**Figure 2:**
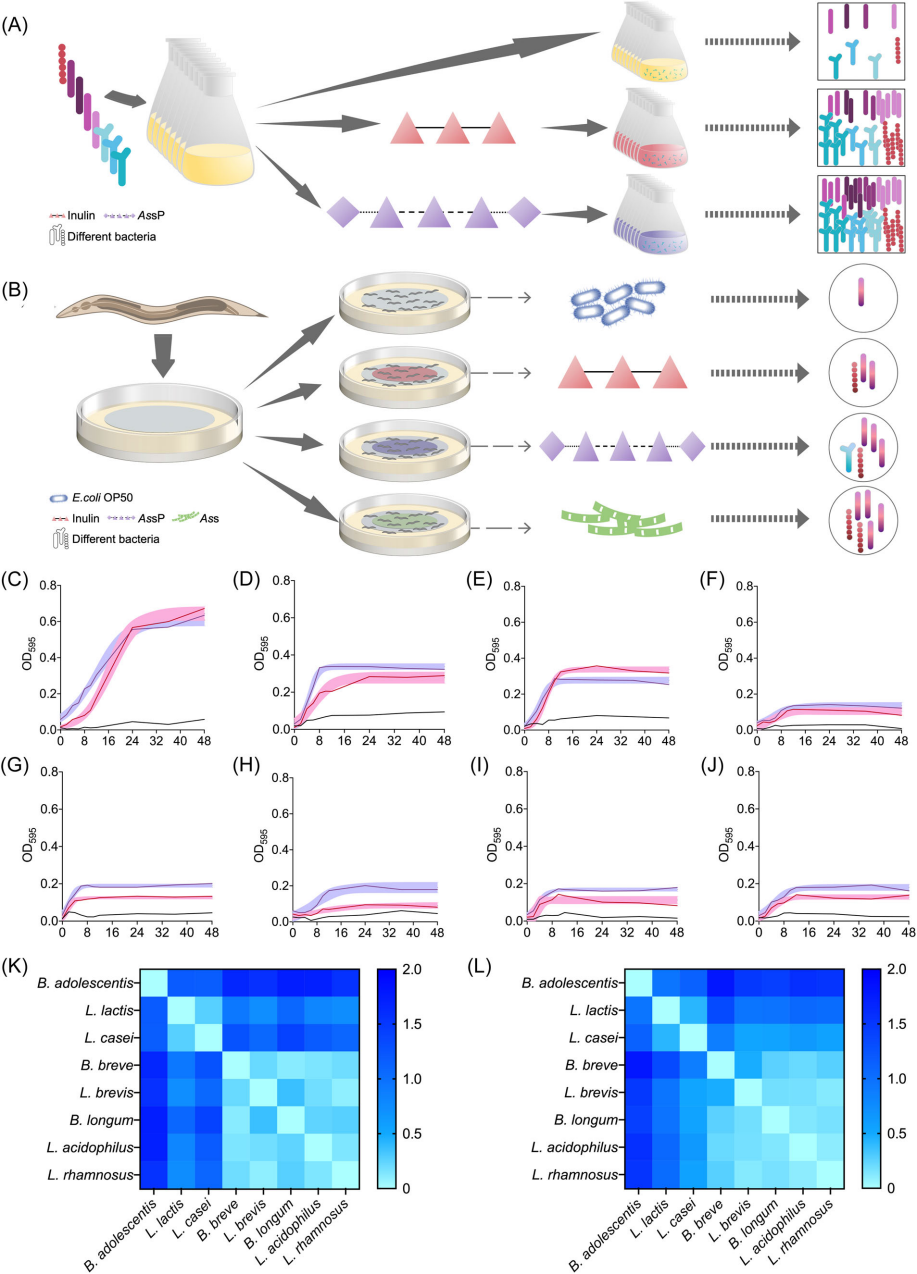
Experimental design and bacterial in vitro growth. (A) Experimental design of in vitro bacterial growth where eight probiotics were individually cultured in the TV broth supplemented with either no carbon source, inulin as the only carbon source, or *Allium schoenoprasum* (*As*sP) as the only carbon source. The OD^595^ of each bacterium at different times was monitored to plot the growth curve. (B) Experimental design of in vivo gut microbiota growth by supplementing *Caenorhabditis elegans* with the nematode growth medium plated with OP50 bacteria (base) or the base medium supplemented with inulin, *As*sP, or *As*s. The worms were grown on different dietary fibers for 15 days before being collected, surface‐cleaned, and sequenced for microbiota analysis. (C–J) Growth curve of *Bifidobacterium adolescentis* ATCC 15703 (C), *Lactococcus lactis* CICC 23609 (D), *Lactobacillus casei* ATCC 334 (E), *Bifidobacterium breve* ATCC 15700 (F), *Lactobacillus brevis* CICC 6239 (G), *Bifidobacterium longum* ATCC 15707 (H), *Lactobacillus acidophilus* ATCC 4356 (I), and *Lactobacillus rhamnosus* ATCC 53103 (J). These probiotics are supplied with no additional carbon source (black), inulin (red), or *As*sP (purple). The background indicates 95% of confidence. (K) and (L) Heatmap showing the dynamic time warping correlation between each growth curve provided in the carbon source of inulin (K) or *As*sP (L).

In the following, eight strains of probiotic bacteria were selected and cultured in a medium where either inulin or *As*sP was offered as the only carbon source or TV broth with no carbon source as the control group (Figure 2A). The growth of each bacterium was monitored. Additionally, in vivo experiment was performed by providing *C. elegans* with either inulin, *As*sP, or *As*s on the basic NGM as the dietary fiber supplement and the NGM medium with no carbon source (base) as the control group. The gut microbiota of *C. elegans* was then sequenced to monitor their dynamics with different supplementation (Figure 2B).

For the in vitro growth experiment, as shown in Figure 2C–J, there was no profound growth of any probiotic bacteria in the absence of carbohydrates in the TV broth. However, all of them could grow in the presence of either inulin or *As*sP with different stationary biomass and growth rates (Supporting Information: Table S2). Among the eight probiotic bacteria studied, five of them (*Bifidobacterium breve* ATCC 15700, *Bifidobacterium longum* ATCC 15707, *Lactobacillus brevis* CICC 6239, *Lactobacillus rhamnosus* ATCC 53103, and *Lactococcus lactis* CICC 23609) yielded both higher biomass and faster maximum growth rates when *As*sP was used as the sole carbon source than inulin. These results indicate that *As*sP supported more bacterial growth than inulin in vitro.

Growth patterns of these eight probiotics with different carbon sources were analyzed and we found that these eight probiotics clustered separately once inulin was used as the sole carbon source. *Bifidobacterium adolescentis* clustered by itself, *L. lactis* and *Lactobacillus casei* clustered, and the rest of them clustered (Figure 2K). Contrastingly, once *As*sP was used as the sole carbon source, clustering between all eight probiotics became indistinctive. *B. adolescentis* clustered by itself and all of the rest clustered (Figure 2L). These results indicate that *As*sP catered more probiotics in their growth that the dynamic time warping (DTW) clustering cannot clearly distinguish their growth patterns, while the utilization of the simple dietary fiber inulin resulted in distinctive growth for these bacteria [32].

### Bacterial genome‐coded degradation of dietary fiber

**Figure 3:**
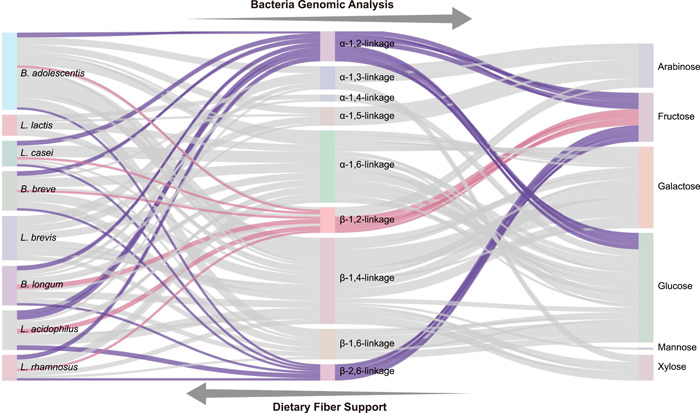
Dietary fiber chemical structures and genomic analysis of their supported bacteria. Left column: eight typical probiotics, from top to bottom are *Bifidobacterium adolescentis* ATCC 15703, *Lactococcus lactis* ATCC 19435, *Lactobacillus casei* ATCC 334, *Bifidobacterium breve* ATCC 15700, *Lactobacillus brevis* ATCC 14869, *Bifidobacterium longum* ATCC 15707, *Lactobacillus acidophilus* ATCC 4356, and *Lactobacillus rhamnosus* ATCC 53103. Middle column: the CAZy reads that can hydrolyze corresponding glycosidic linkages, from top to bottom are α−1,2‐linkage, α−1,3‐linkage, α−1,4‐linkage, α−1,5‐linkage, α−1,6‐linkage, β−1,2‐linkage, β−1,4‐linkage, β−1,6‐linkage, and β−2,6‐linkage. Right column: the CAZy reads that can metabolize corresponding monosaccharide components, from top to bottom are arabinose, fructose, galactose, glucose, mannose, and xylose. The width of the flow indicates the number of reads. From left to right is the genomic analysis of bacteria of the dietary fiber they can metabolize, and from right to left is the support of specific bacteria growth by dietary fiber with a certain chemical structure. The pink flow indicates genes involved in utilizing inulin and the purple flow indicates genes involved in utilizing *Allium schoenoprasum* (*As*sP).

The previous experiment has identified inulin and *As*sP chemical structures, and the relationship between dietary fiber chemical structure and their supported bacterial growth could be established based on this information. Since bacterial growth is coordinated by interwinding of its genome and the growth medium, we analyzed the whole genome of the eight probiotics to find the relation between them [33]. It has to be clarified that the genomes of the *L. lactis* and *L. brevis* analyzed here were not exactly those in the test tube since they are not reported. The same species with known genomes were analyzed instead. Genes coding glycoside hydrolases in each bacterium were analyzed by their function in two aspects: the monosaccharide component they can utilize and the glycosidic linkers joining these monosaccharides they can break (Figure 3). The results show that, for example, *B. adolescentis* genome encodes enzymes that break α−1,3‐, α−1,5‐, and β−1,2‐linked arabinose residues, α−1,2‐, β−1,2‐, and β−2,6‐linked fructose residues, α−1,6‐ and β−1,4‐linked galactose residues, α−1,2‐, α−1,6‐, and β−1,6‐linked glucose residues, β−1,4‐linked mannose residues, and β−1,4‐linked xylose residues. Meanwhile, Figure 3 from the right to left tells us the dietary fiber chemical structure and corresponding bacterial species that the fiber can be metabolized. Fructose connected via the β−1,2‐linker could be viewed as a simplified model of inulin, while fructose connected via the α−1,2‐ and β−2.6‐linker and glucose connected via the α−1,2‐linker could be recognized as part of the *As*sP. For *B. adolescentis*, the numbers of genes supporting growth on inulin are 1, while the numbers of genes supporting growth on *As*sP are 3. For *L. casei*, *B. breve*, *B. longum*, *Lactobacillus acidophilus*, and *L. rhamnosus*, these numbers turned out to be 1 and 3, 1 and 3, 1 and 3, 2 and 6, and 1 and 3, respectively. Apparently, for every bacterium, there are more genes supporting bacterial growth on *As*sP than inulin as the sole carbon source. For example, *B. adolescentis* and *B. longum* are capable of hydrolyzing eight different types of glycosidic linkages, while *L. lactis* is only capable of utilizing four different types. Reversely, a monosaccharide connected via a certain glycosidic bond could meet the genetic requirement of being hydrolyzed by one bacterium, but not necessarily another.

Additionally, the CAZyme (carbohydrate active enzyme) annotation of each bacterial genome was performed with the dbCAN meta server and analyzed in parallel with the fitting parameters from the growth curves of the eight probiotics (Supporting Information: Table S2). It can be seen that the total CAZyme numbers and glycoside hydrolase (GH) numbers are roughly in a positive correlation with the bacterial biomass in the stationary phase, except *B. breve* (Supporting Information: Figure S2A). Further examination of the GH found that families 13, 43, 1, 3, and 25 are the major GH in the probiotic genome (Supporting Information: Figure S2B).

### Complex dietary fiber induced complex worm microbiota growth in vivo

**Figure 4:**
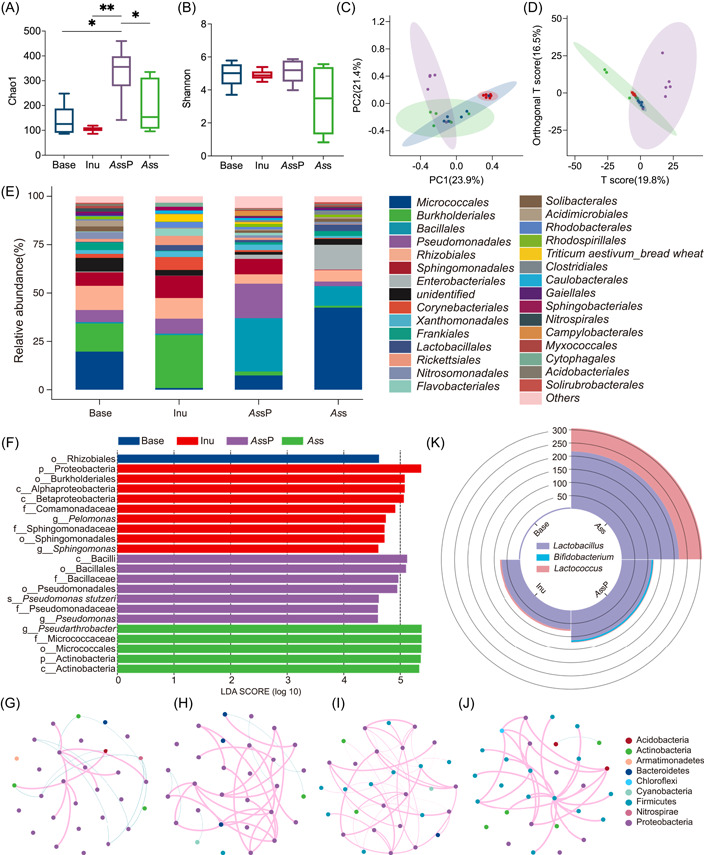
Microbiome analysis of *Caenorhabditis elegans* fed with different dietary fibers. (A) α‐diversity analysis of microbiota richness (Chao1 index), (B) Shannon index, (C) the unweighted_unifrac PCoA analysis, and (D) OPLS‐DA analysis of worm groups (*n* = 6) fed with different carbon sources. The base is the worm fed with OP50‐plated nematode growth medium (NGM); Inu is the worm fed with inulin‐supplemented OP50‐NGM medium; *Allium schoenoprasum* polysaccharide (*As*sP) is the worm fed with *As*sP‐supplemented OP50‐NGM medium; and *As*s is the worm fed with *As*s‐supplemented OP50‐NGM medium. In (C) and (D), blue indicates the base group, red indicates the Inu group, purple indicates the *As*sP group, and green indicates the *As*s group. For the Kruskal–Wallis test, *0.01 < *p* < 0.05 and **0.001 < *p* < 0.01. (E) is the taxonomic distribution of worm microbiota at the order level. (F) LDA score of LEfSe analysis performed at LDA score > 4.6. (G–J) are the microbial co‐occurrence networks of worms fed with the base (G), inulin (H), *As*sP (I), and *As*s (J). Only correlations with Spearman's coefficients < −0.8 or > 0.8 and adjusted *p* values < 0.05 are displayed. The color of nodes represents its phylum. Red and blue lines indicate the positive and negative correlations, respectively. The size of the lines represents their correlation strength. (K) Number of OTUs in the genus *Lactobacillus*, *Bifidobacterium*, and *Lactococcus* found overlapped in the in vitro and in vivo experiments.

Worms on the base medium (OP50 lawn on the NGM), or supplemented with inulin, *As*sP, or *As*s were allowed to grow for 15 days before surface‐cleaned and subjected to gut microbiome analysis. Since the majority of bacteria in worm guts were *E. coli* OP50, OTUs corresponding to *E. coli* were removed before analysis. Compared with worms fed on the base, the observed OTUs did not change significantly in worms fed with inulin (Supporting Information: Figure S3A, Kruskal–Wallis test, *p* > 0.05). Worms fed with *As*sP exhibited increased OTUs (Supporting Information: Figure S3A, 0.001 < *p* < 0.01). α‐analysis with the Chao1 index revealed that inulin did not alter the worm gut microbiota richness, while worm fed with *As*sP exhibited a significant increase in the gut microbiota richness compared with that of the base medium (Figure 4A, Kruskal–Wallis test, *p* < 0.05) and inulin (Figure 4A, Kruskal–Wallis test, 0.001 < *p* < 0.01). However, worm fed with *As*s significantly decreased their gut microbiota richness compared with that fed on *As*sP (Figure 4A, Kruskal–Wallis test, *p* < 0.05). The insignificant variance of the Shannon index and Simpson index among these groups indicated the relatively stable microbial diversity and evenness among worms supplied with different dietary fibers (Figure 4B, Supporting Information: Figure S3B). Unweighted UniFrac distances of 16S rRNA clustering, as a measure of β‐diversity, indicated an almost overlap between worms fed on the base medium and those with inulin supplementation (Figure 4C). On the other hand, worms fed with *As*sP clustered separately from that fed on the base, with a little overlap. And worms fed with *As*s clustered overlapping with both those fed on the base and *As*sP. This is consistent with more β‐diversity measured by Bray–Curtis and weighted_unifrac PCoA analysis (Supporting Information: Figure S3C,D). Orthogonal partial least squares discriminant analysis (OPLS‐DA, SIMCA14, Umetrics, Malmö, Sweden) was used to identify discriminating variables responsible for the clustering patterns among these groups. T score in the x‐axis indicated that there were no significant differences between worms fed with base, inulin, and *As*s, while worms fed with *As*sP and *As*s are deviating from each other (Figure 4D).

The bacteria composition at the order level is similar in worms fed with base and inulin (Figure 4E), except that the relative abundance of *Burkholderiales* increased (one‐way ANOVA, *p* < 0.05) in worms supplemented with inulin. On the other hand, worms fed with *As*sP and *As*s exhibited different bacterial compositions than those with base and inulin supplementation. Worms grown with *As*sP supplementation exhibited a significant increase in the relative abundance of *Bacillales* (one‐way ANOVA, *p* < 0.05), while those grown with *As*s supplementation exhibited a relative abundance increase in *Micrococcales* (one‐way ANOVA, *p* < 0.05) and a decrease in *Sphingomonadales* (one‐way ANOVA, *p* < 0.05). Similarly, on the family level, worms grown on the base and supplemented with inulin exhibited close bacterial composition (Supporting Information: Figure S3E), except the relative abundance of *Comamonadaceae* increased (one‐way ANOVA, *p* < 0.05). LEfSe result identified 22 discriminative features (LDA score > 4.6) whose relative abundance varied significantly between groups (Figure 4F). In the inulin‐supplemented group, the relative abundance of Alphaproteobacteria class, Betaproteobacteria class, Burkholderiales, *Ruminococcaceae*, and *Lactobacillus* increased, consistent with the previous studies in mice [34, 35].

### Complex dietary fiber induced more worm microbiota interaction in vivo

The top 30 OTUs enriched in each worm group were subjected to network analysis to identify their interactions. In worms fed on the base, only 17 OTUs participated in the interactions with 8 positive correlations and 9 negative correlations (Figure 4G). In worms supplemented with inulin, OTUs involved in the interactions increased to 25 with 24 positive interactions and 6 negative interactions (Figure 4H). Once worms were supplemented with *As*sP, OTUs involved in their interactions further increased to 29 and all of the 43 interactions are positive (Figure 4I). However, in worms supplemented with *As*s, OTUs involved in the interactions are 25 with 27 positive correlations and 1 negative correlation (Figure 4J).

When we compare the in vitro probiotic growth and the in vivo worm gut microbiota dynamics supplemented with different dietary fiber, it is found that the genus *Lactobacillus* and *Lactococcus* increased in worms supplemented with inulin compared with worms fed on the base medium (Figure 4K). In worms supplemented with *As*s, the genus *Lactobacillus* and *Lactococcus* increased more significantly. While in worms supplemented with *As*sP, not only the genus *Lactobacillus* and *Lactococcus* increased but also the genus *Bifidobacterium* increased. It is noticed that these three genera, *Lactobacillus, Lactococcus*, and *Bifidobacterium*, cover all of the bacterial genera studied in the in vitro probiotic growth experiment.

## DISCUSSION

Inulin as microbiota‐accessible carbohydrates (MACs) is a clear, simple chemical structure as linear (2 → 1)‐linked β‐d‐fructosyl units attached to the fructosyl moiety of sucrose [36, 37]. Here, we have identified that *As*sP is twofold more complex than inulin: the monosaccharide composition and the diversity of their linkages. Although our NMR experimental setup did not allow us to unambiguously identify the atomic structure of *As*sP, current results could be interpreted with confidence that this dietary fiber is composed majorly of fructose and glucose, via the α(1 → 2) and β(2 → 6) linkages. Thus, the application of these two dietary fibers, with distinct chemical structures, to both the bacterial growth in vitro and in vivo could potentially provide us the information on the relationship between dietary fiber and gut microbiota.

Bacteria elaborate thousands of different enzyme combinations for glycan degradation; genetic variance dictates that different bacteria prefer one carbon source over another [38]. Our in vitro data suggested that *As*sP catered more bacteria growth than inulin as determined by each bacterial intrinsic genome, while our in vivo data suggested a relatively stable diversity and evenness of gut microbiota in *C. elegans* supplemented with different dietary fibers. This is consistent with the finding that high‐fiber supplementation increased microbiome‐encoded CAZymes but not the microbiota diversity [39, 40]. Additionally, the worm gut microbiome richness increased in the presence of *As*sP than inulin. In the inulin‐supplemented group, the relative abundance of class Alphaproteobacteria, class Betaproteobacteria, order Burkholderiales, family *Ruminococcaceae*, and genus *Lactobacillus* increased, which is consistent with previous studies in mice [34, 35]. In the *As*sP‐supplemented group, not only the genus *Lactobacillus* and *Lactococcus* increased but also the genus *Bifidobacterium* increased. These three genera significantly increased in vivo are also of the same genus studied in vitro, suggesting that the in vivo bacterial growth is also fundamentally affected by the chemical structures of the dietary fiber they can encounter, as the same as the in vitro situation [41, 42]. It is worth noting that although different hosts were mentioned here, this process happens between the dietary fiber and the gut bacteria, and no host factors were involved. Thus, we conclude here that the more complex the fiber chemical structure, the more bacteria could grow with which as the MAC.

When the probiotics were cultured in vitro, there was only one bacterium in the test tube and there was no bacterial interaction. However, in the in vivo experiment in the worm gut, there are apparently multiple bacterial interactions. There are more bacterial interactions in the gut of worms supplemented with *As*sP than those supplemented with the base and inulin. Additionally, in the gut of worms fed with the base and inulin, there is a high proportion of positive and frequent negative interactions. On the contrary, there are prevalent positive interactions between the ecological members in the gut of worms supplemented with *As*sP. Positive interaction often originates from metabolites that are utilized by a community member or detoxification of the environment [43]. For example, extracellular digestion of inulin was found to increase the fitness of *Bacteroides ovatus* owing to reciprocal benefits when it feeds other gut species such as *Bacteroides vulgatus* [44]. Thus, even for bacteria that cannot directly utilize dietary fiber, as community members, they can still benefit from transformations of dietary fiber into substrates that can be utilized by them as recipient species [45]. For dietary fiber with a more complex chemical structure, the more bacteria it caters to, the more recipient species raises as indicated by the increased positive interactions.

Precision modulation of gut microbiota has been proposed for an ambitious health and medicinal purposes [46, 47, 48]. The basic idea is to rebalance the microbial community in the gut via all possible methods, including dietary fiber intake as the most simple and convenient way. However, the bottleneck is not the elucidation of microbe species that affect health but the relation between types of dietary fibers and their related gut bacterial dynamics. That is, how discrete dietary fiber chemical structures impact the dynamics of the host microbiome [49, 50]. Inulin, one of the most widely used dietary fibers, could induce multinodular hepatocellular carcinoma in TLR5‐deficient mice marked by an increased abundance of Proteobacteria, which serves as a potential diagnostic signature of dysbiosis and related diseases [51, 52]. In our experiment, the relative abundance of Proteobacteria in the inulin‐supplemented group increased and in the *As*sP‐supplemented group decreased compared with that in the base group. Dietary pectin ameliorates chronic colitis by increasing the relative abundance of Firmicutes, and our *As*sP‐supplemented group yielded a higher increase in Firmicutes abundance than inulin [53]. All the above evidence shows that *As*sP functions better than inulin in vivo by catering to more bacterial growth with its complex structure. Here, with the first step elucidating the relation between the chemical structure of dietary fiber and its regulated gut microbiota dynamics, food, nutritional supplements, and medical intervention on gut microbiota could be applied with primary theoretical guidance.

## CONCLUSION

In conclusion, genomic analysis of individually cultured bacteria indicated that the more complex the dietary fiber utilized as the carbon source, the more bacteria it could possibly support. And this dietary fiber chemical structure–bacteria growth relation is validated in the *C. elegans* animal model, provided that the microbiota community interactions and gene transfer scenario are considered. This study lays a theoretical foundation for the food, nutritional supplements, and medical intervention of gut microbiota.

## METHODS

### Materials

Inulin from dahlia tubers (I811905‐250 g, CAS # 9005‐80‐5) was purchased from Macklin Inc. (Shanghai, China). The stalks of *A. schoenoprasum* and *As*sP were obtained as previously described [30]. Strains of *B. adolescentis* ATCC 15703, *B. breve* ATCC 15700, *B. longum* ATCC 15707, *L. acidophilus* ATCC4356, *L. brevis* CICC 6239, *L. casei* ATCC 334, *L. rhamnosus* ATCC 53103, and *L. lactis* CICC 23609 were purchased from BeNa Culture Collection.

### Nuclear magnetic resonance analysis of *As*sP

The *As*sP was dissolved in 3% (w/w) D_2_O and incubated for 3 h before the solution was freeze‐dried. This step was repeated three times. The deuterated *As*sP was dissolved again in D_2_O of about 100 mg/ml for NMR measurement. All NMR spectra were acquired at 850 (^1^H) or 212 MHz (^13^C) on a Bruker NMR instrument 850 MHZ spectrometer using a 5 mm CPTCI probe at 27°C. One‐dimensional proton, ^13^C and two‐dimensional COSY, edit‐HSQC, HSQC‐TOCSY, NOESY, HMBC, and H2BC spectra were acquired. 1‐D proton spectrum was averaged from 16 scans, and 1‐D ^13^C spectrum was acquired in 2048 scans. The 2‐D COSY spectra were acquired in four scans per increment and 256 increments, the edit‐HSQC spectra in 8 scans and 256 increments, the HSQC‐TOCSY spectra in 16 scans per increment and 256 increments, the NOESY spectra in 8 scans per increment and 256 increments, and the HMBC spectra in 32 scans and 128 increments. The relaxation delays for the NMR experiments were 2 s. Mixing times for the TOCSY and NOESY spectra were 80 and 300 ms, respectively.

### In vitro bacteria growth on different carbon sources

The four *Lactobacillus* strains were activated in MRS broth at 37°C for 16 h three times anaerobically, and the three *Bifidobacterium* were activated with MRS broth containing 0.5% cysteine at 37°C for 16 h three times anaerobically. These above strains were cultured in anaerobic vials charged with nitrogen. The *Lactococcus lactis* were activated in MRS broth at 37°C for 16 h aerobically (three times). Each bacterium was collected by centrifugation at 5000 rpm for 20 min, washed with PBS buffer containing 5% cysteine, and centrifuged again to remove the residual MRS broth. Each bacterium was suspended with a tryptone‐vitamin base (TV) medium, diluted to OD^595^ of 1.0, and added to a final volume of 40 ml TV medium before being cultured at 37°C for 48 h. The TV broth was supplemented with 0.5% inulin, 0.5% *As*sP, and no dietary fiber. A syringe was used to sample 200 μL of culture medium through the sealing gasket at 0, 2, 4, 6, 8, 10, 12, 24, 36, and 48 h. The absorption at 595 nm was recorded on a plate reader (SpectraMax M2^e^; Molecular Devices), and the experiments were performed in triplicates.

### In vitro bacteria growth data analysis

The maximum growth rates of each above strain under different carbon sources were calculated by fitting the growth curve to the logistic growth model with GraphPad Prism (version 8.3.0). The 95% confidence interval of each growth curve was also plotted as the shaded region. The growth curves of eight typical probiotics were clustered with the DTW method with Python 3.9.5 [32]. The DTW distance obtained from the above method was plotted into a heat map with GraphPad Prism.

### Genomic analysis of the probiotics

The genomic information of *B. adolescentis* ATCC 15703, *B. breve* ATCC 15700, *B. longum* ATCC 15707, *L. acidophilus* ATCC 4356, *L. casei* ATCC 334, and *L. rhamnosus* ATCC 53103 were acquired from the American type culture collection (ATCC, https://genomes.atcc.org/genomes). Among these bacteria, the information of *L. brevis* CICC 6239 and *L. lactis* CICC 23609 could not be found and was replaced with that of *L. brevis* ATCC 14869, *L. lactis* ATCC 19435. For each probiotic, the genome annotation was examined and all the glycosidase with EC number was inquired in The Comprehensive Enzyme Information System (BRENDA, https://www.brenda-enzymes.org/) for the glycosidic linkage and monosaccharide component that each glycosidase could hydrolyze. The collected information was plotted into the Sankey diagram with R v.1.3.1093. The genome of each probiotic was annotated with the dbCAN meta server (http://bcb.unl.edu/dbCAN2/) to get the CAZyme domain information. All the CAZyme information for each probiotic was integrated and plotted with GraphPad Prism [54].

### In vivo worm microbiota growth on different carbon sources

Inulin and freeze‐dried *As*sP were dissolved in sterilized water to get a 100 mg/ml solution; meanwhile, the dried chive stalk was ground, filtered through a 200‐mesh screen, and mixed with sterilized water to get a 100 mg/ml suspension. These three solutions were sterilized in an autoclave, spread on the NGM with *E. coli* OP50, and incubated at 37°C for 5 h. The synchronized wild‐type N2 *C. elegans* were cultured on the NGM with only OP50 (base), base supplemented with inulin, base supplemented with *As*sP, base supplemented with *As*s for 3–4 days, and transferred to another NGM (with or without dietary fiber supplementation) before the dietary fibers were exhausted. After 15 days’ cultivation on each supplementation, the adult *C. elegans* at about the same growth stage were collected, washed three times with M9 buffer, and inoculated onto a plain NGM with 100 μg/ml gentamicin for 1 h to remove the contaminant bacteria on the surface, washed again with sterilized water, and stored at −80°C for microbiome sequencing.

### 16S rRNA gene sequencing and analysis

The *C. elegans* microbiota was sequenced according to [55] with modifications. The nematode gut microbiota DNA was extracted with the MO BIO PowerSoil DNA isolation kit (No. 12888; MO BIO Laboratories, Inc.) according to the manufacturer's instructions. Primers (F: 5'‐GTACTCCTACGGGAGGCAGCA‐3' and R: 5'‐GTGGACTACHVGGGTWTCTAAT‐3') with barcodes were used to amplify the bacterial 16S rRNA gene V3–V4 region. The polymerase chain reaction (PCR) contained 30 ng of extracted DNA, 0.4 μM of each primer, and 12.5 μl of 2× Taq Plus Master Mix (Tsingke Biological Technology), water was added to a final volume of 25 μl, and the reaction was carried out as follows: 94°C for 30 s, 50°C for 30 s, 72°C for 60 s, 25 cycles. PCR was carried out in triplicate, combined, gel‐purified with 2% agarose gel, and extracted with the AxyPrep DNA Gel Extraction Kit (AP‐GX‐250G; Corning). The library was sequenced with Illumina MiSeq PE300 (Illumina) at Allwegene Technologies Inc.

The 16S rRNA gene sequencing data was filtered, trimmed, and classified into operational taxonomic units (OTUs) at a 97% similarity cutoff with Trimmomatic (v 0.33) [56]. The taxonomy of each OTU was assigned taxonomy with the SILVA database (Release 128, http://www.arb-silva.de). The α‐diversity analyses, including Chao1, observed_OTUs, Shannon index, Simpson index, and the Kruskal–Wallis test results, were obtained via Qiime2 analysis. The Bray–Curtis PCoA analysis, Unifrac PCoA analysis (including unweighted and weighted), OPLS‐DA analysis, LEfSe analysis (with LDA score set at 4.6), and network analysis were performed with the Microeco bioinformatics cloud (https://www.bioincloud.tech/, Microeco Tech Co.). Interaction networks were constructed with Gephi v.0.9.2 [57].

### Statistical analysis

Data were tested for normality of distribution and equality of variance using the Shapiro–Wilk tests and Bartlett's tests, respectively. If normally distributed and homogeneous, data were analyzed by one‐way analysis of variance followed by Duncan's test to assess the statistical significance of the differences between groups. When data were not normally distributed or nonhomogeneous, data were analyzed using a Kruskal–Wallis test followed by Dunn's multiple range posttest. *p* < 0.05 were considered significant. All statistical analyses were performed by Prism 9.0 (GraphPad Software).

## AUTHOR CONTRIBUTIONS

**Xin Meng**: Investigation; formal analysis; visualization. **Jun Zheng**: Investigation; visualization. **Fengqiao Wang**: Investigation. **Jie Zheng**: Methodology; Writing – review & editing. **Dong Yang**: Conceptualization; supervision; funding acquisition; writing – original draft; writing – review & editing. All authors contributed to discussions on the results and approved the manuscript.

## CONFLICT OF INTEREST

The authors declare no conflict of interest.

## Supporting information

Supplementary information.

Supplementary information.

## Data Availability

Sequences generated in this study are stored in the National Center for Biotechnology Information (NCBI) and the project number is PRJNA748876. All the sequencing data have been deposited in NCBI under the BioProject accession number PRJNA748876 https://www.ncbi.nlm.nih.gov/bioproject/PRJNA748876 and in GSA under the BioProject accession number PRJCA012863 https://ngdc.cncb.ac.cn/gsa/browse/CRA008685. The data and scripts used are saved at https://github.com/MencyX/Dietary-fiber-chemical-structure-determined-gut-microbiota-dynamics. Supplementary materials (figures, tables, scripts, graphical abstract, slides, videos, Chinese translated version, and updated materials) may be found in the online DOI or iMeta Science http://www.imeta.science/.
