# Supplementary material for: Dietary fiber chemical structure determined gut microbiota dynamics
Source: Imeta. 2022 Nov 28;1(4):e64. doi: 10.1002/imt2.64 (PMC10989905; doi:10.1002/imt2.64)
Supplement: Supplementary file 1 — Supplementary information. [file IMT2-1-e64-s002.docx]

**Dietary Fiber Chemical Structure Determined Gut Microbiota Dynamics**

Xin Meng^1,†^, Jun Zheng^1,†^, Fengqiao Wang^1^, Jie Zheng^2^ and Dong Yang^1,*^

^1^Beijing Key Laboratory of Functional Food from Plant Resources, College of Food Science & Nutritional Engineering, China Agricultural University, Beijing 100083, China

^2^Center for Food Safety and Applied Nutrition, U.S. Food and Drug Administration, College Park MD 20740, USA

Correspondence should be addressed to Dong Yang [dyang@cau.edu.cn](mailto:dyang@cau.edu.cn)

^†^These authors contributed equally to this work

This file includes:

Figure S1 Supplementary NMR spectrum of deuterated *As*sP in D_2_O.

Figure S2 The CAZyme annotation of the prebiotics involved in this study.

Figure S3 Further Microbiome analysis of *C. elegans* fed with different dietary fiber.

**
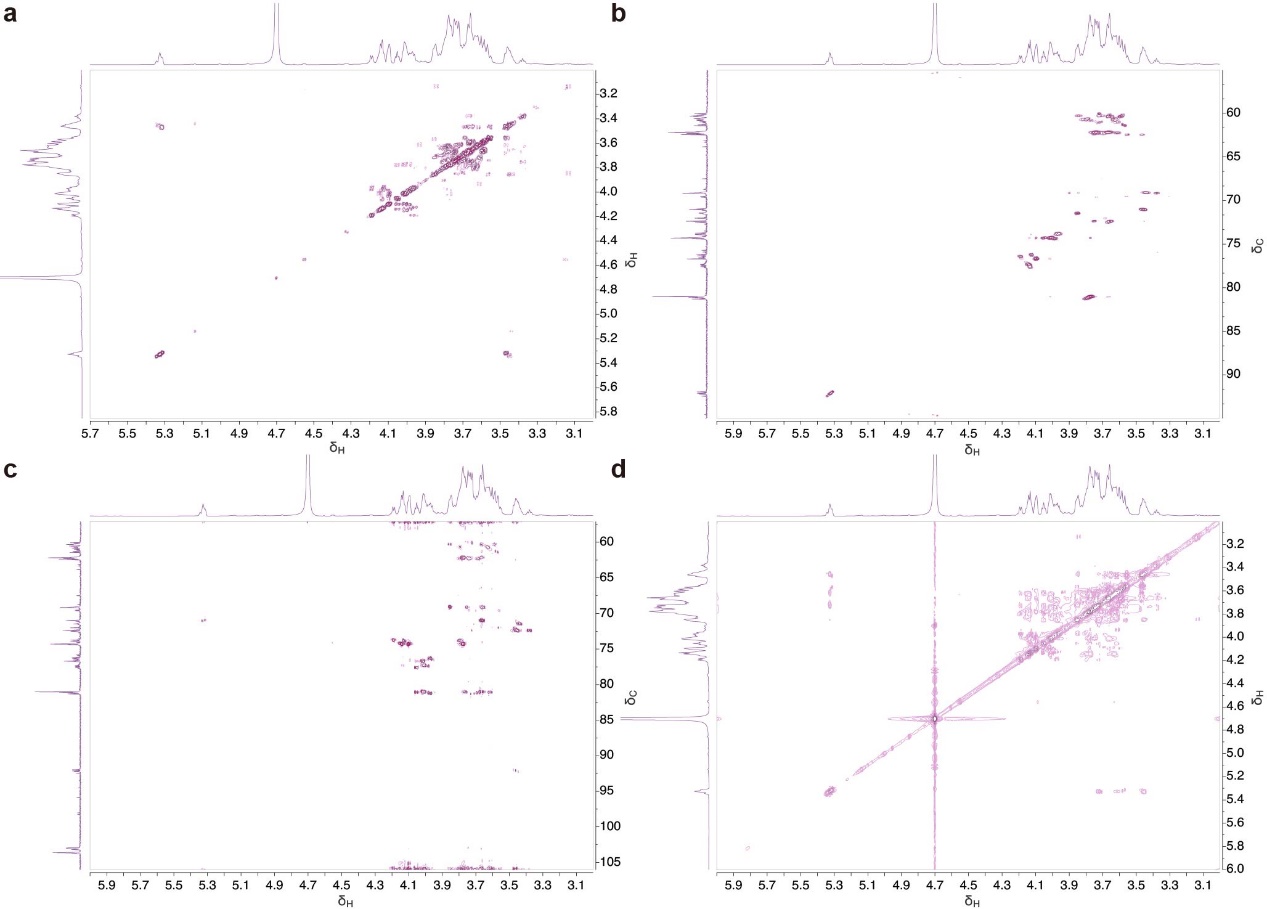
**

**Figure S1 Supplementary NMR spectrum of deuterated *As*sP in D_2_O. a**, The COSY NMR spectrum of deuterated *As*sP in D_2_O. **b,** the HSQCED NMR spectrum of deuterated *As*sP in D_2_O. **c,** The H2BC NMR spectrum of deuterated *As*sP in D_2_O. **d,** The NOESYPHER NMR spectrum of deuterated *As*sP in D_2_O.

**
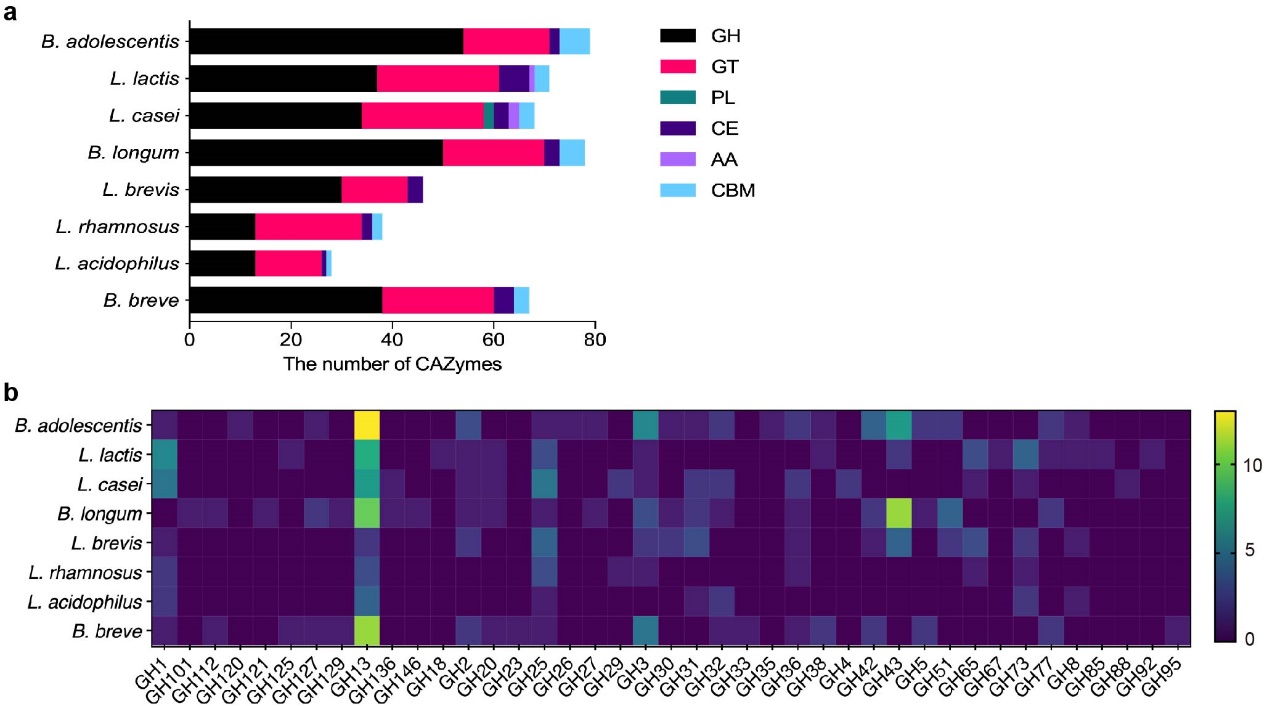
**

**Figure S2 The CAZyme annotation of the prebiotics involved in this study. a,** the CAZyme annotation of the genomes of eight probiotics involved in this study, including the glycoside hydrolases (GH), glycosyltransferases (GT), polysaccharide lyases (PL), carbohydrate esterase (CE), auxiliary activities (AA), and carbohydrate binding modules (CBM). **b**, the detailed number of genes involved in each GH families of the eight probiotics involved in this study.

**
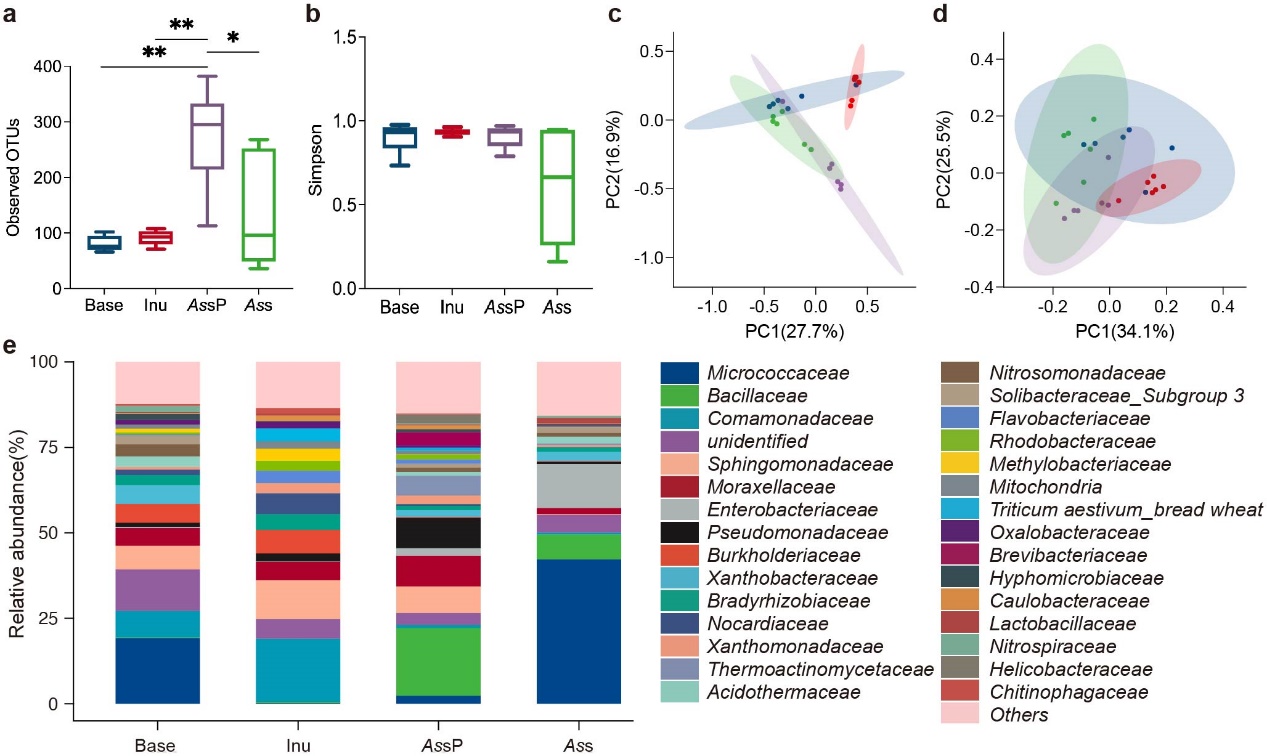
**

**Figure S3 Further Microbiome analysis of *C. elegans* fed with different dietary fiber. a** observed_OTUs, **b** Simpson index, **c** The Bray-Curtis PCoA analysis, and **d** the weighted_unifrac PCoA analysis of worm groups (n=6) fed with different carbon sources. In **c** and **d**, blue indicate base group, red indicates Inu group, purple indicates *As*sP group, and green indicates *As*s group. Base is the worm group fed with OP50 plated NGM medium; Inu is the worm group fed with inulin supplemented OP50-NGM medium; *As*sP is the worm group fed with AssP supplemented OP50-NGM medium; and *As*s is the worm group fed with Ass supplemented OP50-NGM medium. For the Kruskal-Wallis test, * stands for the 0.01< *P* <0.05, and ** stands for that 0.001< *P* <0.01. **e**, the taxonomic distribution of worm microbiota at family level.
